# Antinociceptive tolerance to NSAIDs in the anterior cingulate cortex is mediated via endogenous opioid mechanism

**DOI:** 10.1186/s40360-017-0193-y

**Published:** 2018-01-06

**Authors:** Nana Tsiklauri, Natia Pirkulashvili, Ivliane Nozadze, Marina Nebieridze, Gulnaz Gurtskaia, Elene Abzianidze, Merab G. Tsagareli

**Affiliations:** Lab of Pain and Analgesia, Beritashvili Center for Experimental Biomedicine, 14, Gotua Street, 0160 Tbilisi, Georgia

**Keywords:** Antinociception, Endogenous opioids, Descending modulation, Nociception, Non-opioid tolerance

## Abstract

**Background:**

In the past decade several studies have reported that in some brain areas, particularly, in the midbrain periaqueductal gray matter, rostral ventro-medial medulla, central nucleus of amygdala, nucleus raphe magnus, and dorsal hippocampus, microinjections of non-steroidal anti-inflammatory drugs (NSAIDs) induce antinociception with distinct development of tolerance. Given this evidence, in this study we investigated the development of tolerance to the analgesic effects of NSAIDs diclofenac, ketorolac and xefocam microinjected into the rostral part of anterior cingulate cortex (ACC) in rats.

**Methods:**

Male Wistar experimental and control (saline) rats were implanted with a guide cannula in the ACC and tested for antinociception following microinjection of NSAIDs into the ACC in the tail-flick (TF) and hot plate (HP) tests. Repeated measures of analysis of variance with post-hoc Tukey-Kramer multiple comparison tests were used for statistical evaluations.

**Results:**

Treatment with each NSAID significantly enhanced the TF and HP latencies on the first day, followed by a progressive decrease in the analgesic effect over a 4-day period, i.e., developed tolerance. Pretreatment with an opioid antagonist naloxone completely prevented the analgesic effects of the three NSAIDs in both behavioral assays.

**Conclusions:**

These findings support the concept that the development of tolerance to the antinociceptive effects of NSAIDs is mediated via an endogenous opioid system possibly involving descending pain modulatory systems.

## Background

The most important signaling mechanism for imminent harm is the pain system. Studies of the emotional and motivational basis of pain reveal a diverse and complex set of processes by which the affective experience of pain is realized. In particular, the perception of both pain intensity and aversiveness is the complex process by which the brain constructs the sensory and emotional sensation of pain and challenges any standard “perception-action” model [1].

The first anatomical, physiological and behavioral investigations have demonstrated the important role of the brain limbic system in the affective-motivational component of pain. Some clinical evidence and animal studies have shown the importance of the anterior cingulate cortex (ACC) in affective aspects of pain [2]. Injection of local anesthetics into the lateral hypothalamus, the cingulum bundle and the dentate nucleus of the hippocampus has temporarily blocked neural activity and induced significant analgesia during late tonic phase of pain perception [3–5]. Furthermore, surgical lesions of the cingulate cortex and/or the cingulum bundle were described as able to reduce the emotional but not the sensory component of chronic pain [6]. On the strength of positron emission tomography scan images, it has been concluded that activation of structures associated with autonomic and limbic system functions, such as the insula and the ACC, may reflect the affective aspect of pain experience [7].

Nowadays, it is well known that the ACC is involved in pain perception primarily receiving extensive projections from the medio-dorsal thalamic nucleus and broadly connects with relevant regions of the descending pain modulation system. A projection from the spinal dorsal horn through the medial/intra-laminar thalamic nuclei to the ACC has been proposed to process information on pain-related unpleasantness. In addition to contributing to the immediate affective consequences of noxious stimulation, the ‘ACC system’ may contribute to the avoidance learning that sometimes follows as a secondary reaction to pain. Neurons in the rostral ACC are required for pain-related aversion learning – a process that directly reflects the affective component of pain [8]. Just recently, in chronic pain patients proton magnetic resonance spectroscopy has revealed a different metabolite status in the AAC compared to controls. The mean levels of glutamic acid (Glu)/total creatine and Glu + glutamine/total creatine are higher, but lower for *N*-acetylaspartate/total creatine, compared with healthy controls [9].

Unlike opioid analgesics, non-steroidal anti-inflammatory drugs (NSAIDs) are the most widely used analgesics in the treatment of mild, not severe pain. For the last years, however, it was established that repeated administration of NSAIDs systemically or in the midbrain periaqueductal grey matter (PAG) induced tolerance to these drugs, similarly to opioid analgesics, and cross-tolerance to morphine [10–16].

We have recently shown that tolerance develops to analgesic effects of the commonly used NSAIDs metamizol, diclofenac, ketorolac and xefocam given intraperitoneally (i.p.) in juvenile and adult rats in models of acute pain [17, 18]. We have also revealed that repeated microinjections of these non-opioids into the dorsal hippocampus (DH), the nucleus raphe magnus (NRM), and the central nucleus of amygdala (CeA), induce antinociception and the effects of tolerance and cross-tolerance to morphine [13, 14, 19–21]. These findings strongly support the suggestion of endogenous opioids involvement in NSAIDs antinociception and tolerance in the descending pain-control system [13, 22].

In the present study, we hypothesized that the analgesic effects of the three NSAIDs, diclofenac, ketorolac and xefocam microinjected into the ACC would exhibit tolerance mediated via endogenous opioids.

## Methods

### Animals

The experiments were performed on male Wistar rats weighing 200 to 250 g at the time of surgery bred at the Beritashvili Exp. BMC. The animals were kept under standard housing conditions (22 ± 2 °C, 65% humidity, and light from 7:00 a.m. to 8:00 p.m.) and kept on a standard dry diet with water freely available. Every procedure was designed to minimize discomfort to animals and efforts were made to minimize their number. Six rats were used for each experimental and control groups. All procedures adhered to the Guidelines of the International Association for the Study of Pain regarding animal experimentation [23] and were approved by Institutional Animal Care and Use Committee of the Beritashvili Experimental Biomedicine Center.

### Surgical procedures

Brain surgery was performed as described previously [19]. In short, under anesthesia with intramuscular administration of ketamine (100 mg/kg, “KharkovPharm”, Ukraine), a 12-mm-long stainless steel guide cannula (Small Parts, Inc., USA) was stereotaxically implanted into the rostral part of ACC (area I) (AP: 2.70; L: +0.5; H: 2.5) according to the coordinates in the atlas of Paxinos and Watson (1997) [24] siting the tip 2 mm above the ACC. The guides were anchored to the cranium by dental cement. The guide cannula was plugged with a stainless steel stylet. Thereafter, the rats were handled every day for 3 days for 15 min to get familiar with the testing protocol and experimental environment. During this time, the stylet was removed and 14 mm-long stainless steel microinjection cannula was inserted into the guide cannula to reach the ACC, but no drug was injected. This helped to habituate the rats to the injection procedure and to reduce artifacts arising from mechanical manipulation during the test days. Five days after surgery the microinjection cannula, attached to a 50-μl Hamilton syringe (Hamilton, Inc., USA), was joined to the guide cannula, and the drug was introduced through it while the rat was gently restrained.

### Drugs

Diclofenac (diclofenac sodium, 75 μg/0.5 μl, Hemofarm, Serbia), ketorolac (ketorolac tromethamine, 90 μg/0.5 μl, Grindex, Latvia) or xefocam (lornoxicam, 12 μg/0.5 μl, Nycomed, Austria) were injected through the microinjection cannula as we used in previous works [19, 20]. The guide cannula was then plugged with a stainless steel stylet. Isotonic saline was injected in the same volume (0.5 μl, GalichPharm, Ukraine) and manner in a separate group of rats for controls. In the second set of experiments a non-selective opioid receptor antagonist naloxone (0.2 μg/0.5 μl, Polfa S.A., Poland) was injected through the microinjection cannula [19]. Solutions were microinjected in about 10–12 s.

### Behavioral testing

Twenty minutes post microinjection of NSAIDs, i.e. 10-min before the peak of the drugs’ effect is normally reached, animals were tested for antinociception using the tail-flick (TF) and hot plate (HP) tests. For the TF test, the distal part of the tail was stimulated with a light beam and the latency measured until the tail was reflexively flicked away from the beam (IITC #33, IITC Life Science, Inc., Woodland Hills, CA, USA). For the HP test, the rat was placed on a 55 °C hot plate and the latency to the first hindpaw licking or jumping was measured (IITC #39). The cut-off time was 20 s for both TF and HP latencies. Each rat was tested with both TF and HP in the same session. A similar procedure was followed for the repeated microinjection of diclofenac, ketorolac, xefocam or saline for four consecutive days. In special control experiments, saline microinjections into the ACC was followed by a non-selective opioid receptor antagonist naloxone (0.5 μl, Polfa S.A., Poland) and tested for TF and HP latencies.

In the second set of experiments, pretreatment of rats with a non-selective opioid receptor antagonist naloxone in the ACC was followed by TF and HP tests. 10 min after they were treated with NSAIDs in the same dose as in the first set of experiments and were then tested again. Different animal groups were used for the first and second sets of experiments.

### Histology

**Fig. 1:**
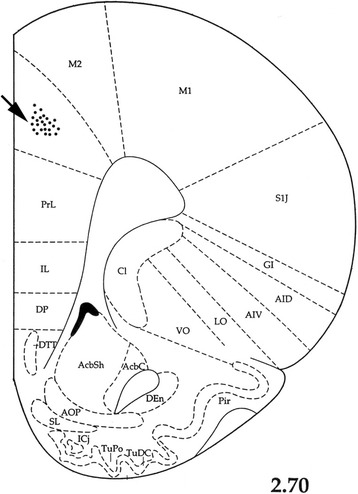
A serial coronal section of the rat brain showing placement of microinjections unilaterally in the ACC (the black arrow). The number to the below of the section represents millimeters relative to bregma, adapted from the Paxinos and Watson (1997) stereotaxic atlas [24]

At the end of each set of experiments, the microinjection sites were marked with 2 μl of saturated solution of Direct Blue-1 (Sigma-Aldrich) and the animal was euthanized with pentobarbital. After fixation by immersion in 10% formalin, the brain was sectioned and counterstained with Cresyl Violet. The microinjection sites were histologically verified and plotted according to Paxinos and Watson (1997) stereotaxic atlas coordinates [24]. Representative microinjection sites are shown in Fig. 1.

### Statistical analysis

All data are presented as mean ± S.E.M. One-way analysis of variance (ANOVA) or repeated measures of analysis of variance (rMANOVA) with post-hoc Tukey-Kramer or Dunnett multiple comparison tests were used for statistical comparisons between treated and saline groups, and treated and naloxone groups, respectively. The Kolmogorov–Smirnov test was applied to verify normality. The statistical software utilized was InStat 3.05 (GraphPad Software, USA). Differences between means of vehicle control and treated groups, and naloxone and treated groups of rats were acknowledged as statistically significant if *P* < 0.05.

## Results

### Tolerance to antinociceptive effects of NSAIDs

**Fig. 2:**
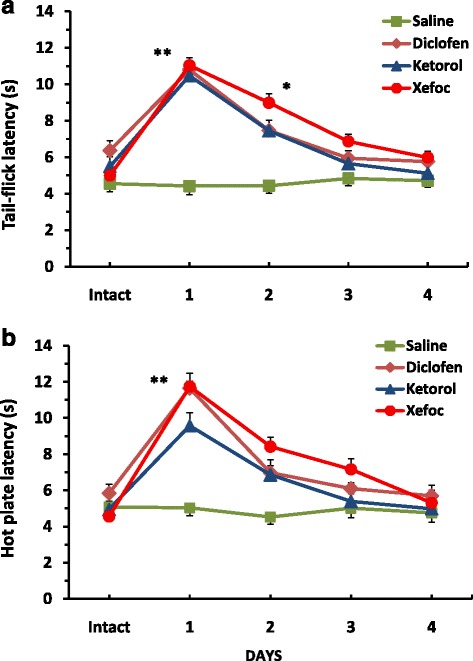
Microinjections of NSAIDs into the ACC for 4 consecutive days result in a progressive decrease in TF (**a**) and HP (**b**) latencies as compared to vehicle saline control. *- *P* < 0.05, **- *P* < 0.01

In the first set of experiments we found that microinjection of NSAIDs into the ACC produced antinociception as detected by a latency increase in TF and HP compared to the baseline control of intact rats and a control group with saline microinjected into the same site as well. The rMANOVA revealed that the TF latency significantly increased for clodifen [F(9, 20) = 24.222, *P* < 0.0001], ketorolac [F(9, 20) = 71.399, *P* < 0.0001], and xefocam [F(9, 20) = 101.13, *P* < 0.0001], respectively, but not for saline group [F(9, 20) = 0.4148, *P* = 0.7955, not significant]. The TF latency differences between NSAIDs treated groups and the saline group by Dunnett test were significant in the first experimental day for diclofenac (*t* = 3.608, *P* < 0.01), ketorolac (*t* = 3.424, *P* < 0.01), and xefocam (*t* = 3.741, *P* < 0.01), respectively (Fig. 2a).

We found similar significant differences in the HP latencies for diclofenac [F(9, 20) = 29.045, *P* < 0.0001], for ketorolac [F(9,20) = 55.307, *P* < 0.0001], and for xefocam [F(9,20) = 90.93, *P* < 0.0001], respectively, but not for saline control [F(9,20) = 1.299, *P* = 0.3123, not significant]. The HP latency differences between NSAIDs treated groups and the control group by Dunnett test were significant in the first experimental day for diclofenac (*t* = 2.687, *P* < 0.05) and xefocam (*t* = 2.728, *P* < 0.05), but not for ketorolac (*t* = 1.846, *P* > 0.05) (Fig. 2b).

Subsequent NSAIDs microinjections caused progressively less antinociception, so by day 4 there was no effect, similar to saline microinjections for both the TF and the HP tests, i.e. induced tolerance. By the second experimental day the TF latency differences between NSAIDs treated groups and the saline group were significant only for xefocam (*t* = 3.066, *P* < 0.05). There were not significant differences between NSAIDs treated groups and the control in the HP test for the second experimental day (Fig. 2).

### Pretreatment with naloxone prevents NSAIDs-induced antinociception

**Fig. 3:**
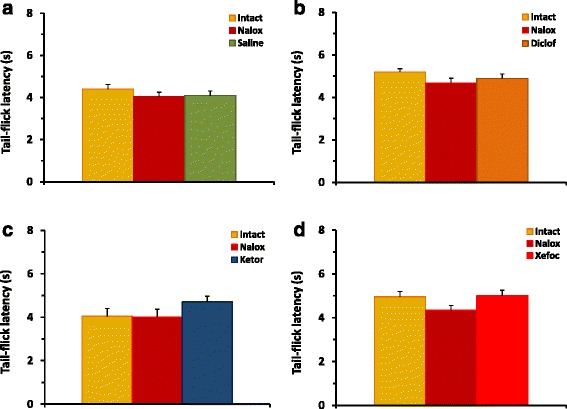
Pretreatment with naloxone before microinjections of NSAIDs into the ACC results in prevention of NSAID-induced antinociception in TF latency for diclofenac (**b**), ketorolac (**c**), and xefocam (**d**), respectively. Control experiments of pretreatment with naloxone before microinjection of saline into the ACC do not significantly change TF latency (**a**)

**Fig. 4:**
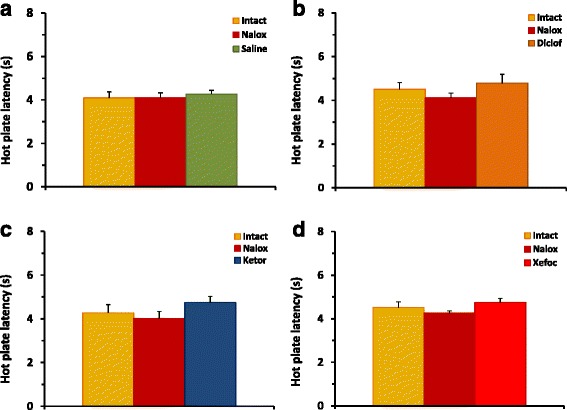
Pretreatment with naloxone before microinjections of NSAIDs into the ACC results in prevention of NSAID-induced antinociception in HP latency for diclofenac (**b**), ketorolac (**c**), and xefocam (**d**), respectively. Control experiments of pretreatment with naloxone before microinjection of saline into the ACC do not significantly change HP latency (**a**)

In the second set of experiments, we tested if pretreatment with a non-selective opioid receptor antagonist naloxone prevents antinociception induced by NSAID microinjected into the ACC. Pretreatment with naloxone completely prevented the analgesic effects of diclofenac, ketorolac, and xefocam in the TF test. The differences between NSAIDs injected and naloxone injected groups are not significant [ANOVA: F(3,32) = 1.419, *P* = 0.2552, not significant] (Fig. 3). The same results are in the HP test for diclofenac, ketorolac, and xefocam, respectively [ANOVA: F(3,32) = 1.829, *P* = 0.1618, not significant] (Fig. 4).

Special control testing with naloxone microinjections into the ACC followed by saline statistically did not change the latency to respond in the TF [ANOVA: F(2,15) = 1.301, *P* = 0.3012, not significant] (Fig. 3a), and HP [ANOVA: F(2,15) = 0.2939, *P* = 0.2939, not significant] tests, respectively (Fig. 4a).

## Discussion

The data reported in this study demonstrate that microinjection of commonly used NSAIDs, diclofenac, ketorolac and xefocam into the rostral part of ACC induces antinociception. These findings are in resemblance with the results of our and other colleagues’ previous investigations in an acute pain model with TF and HP tests, and in which metamizol, xefocam, ketorolac or lysine-acetylsalicylate were given systemically or microinjected into the PAG [10, 11, 16–18, 25], into the CeA [13, 21], and the NRM [13, 20, 26]. In the other investigation, responses of spinal dorsal horn wide-dynamic range neurons of rats to mechanical noxious stimulation of a hindpaw were strongly inhibited by intravenous metamizol [27].

More importantly, repeated administrations of these NSAIDs into the ACC over a period of 4 days resulted in a progressive decrease in antinociceptive effectiveness, i.e. development of tolerance, reminiscent of that induced by opiates [10, 12, 13, 18, 28]. The present data confirm our previous results in which development of tolerance was observed to the analgesic effects of diclofenac, ketorolac and xefocam microinjected into the DH of rats. After injection of each drug, a progressive decrease in TF and HP latency (i.e., tolerance) was noticed over the 4-day period [19, 26].

The mechanism producing tolerance to NSAIDs can be due to the participation of endogenous opioids [13, 22, 29, 30]. Here we clearly showed that pretreatment of a non-selective opioid receptor antagonist naloxone significantly diminishes NSAIDs-induced antinociception. These findings confirm our previous evidence where pretreatment with naloxone prevented antinociceptive effects of metamizol, ketorolac and xefocam in juvenile and adult rats. Moreover, in morphine-tolerant juvenile and adult rats we revealed effects of cross-tolerance to metamizol, ketorolac and xefocam [18]. As stated above, NSAIDs antinociception in the DH was reduced by pre- and post-treatment with naloxone [19, 26]. Just recently we showed that systemic pretreatment with naloxone completely prevented the analgesic effects of NSAIDs (diclofenac, ketorolac and xefocam, *i.p.*) in thermal paw withdrawal (Hargreaves) test and mechanical paw withdrawal (von Frey) test in a chronic inflammatory pain model, the formalin test [31].

These and the present data also confirm previous results that anti-nociception induced by systemic metamizol involves endogenous opioids that can be blocked by naloxone at the levels of the PAG, the NRM and the spinal dorsal horn [32], as well as other findings that endogenous opioids are involved in the potentiation of analgesia observed with a combination of morphine plus dipyrone [33]. These data suggest a role for endogenous opioidergic descending pain control circuits. The latter consists of the brainstem pain modulatory network with critical links in the PAG as well as the rostral ventro-medial medulla [22, 29, 30, 34].

## Conclusions

Similar to previous studies, we have demonstrated that administration of diclofenac, ketorolac and xefocam, widely used non-opioid, NSAID analgesics, into the rostral part of the ACC, induces antinociception in rats. When administered repeatedly, tolerance developed to the antinociceptive effects of these drugs. The present findings support the concept that the development of tolerance to the antinociceptive effects of NSAIDs is mediated via an endogenous opioid system possibly involving descending pain modulatory systems.

## Abbreviations

ACC: The anterior cingulate cortex
ANOVA: The analysis of variance
CeA: The central nucleus of amygdala
DH: The dorsal hippocampus
HP: Hot plate
i.p.: Intraperitoneal
NRM: The nucleus raphe magnus
NSAIDs: Non-steroidal anti-inflammatory drugs
PAG: The periaqueductal grey matter
S.E.M.: Standard error of the mean
TF: The tail-flick
